# Molecular and cytological analysis of widely-used Gal4 driver lines for *Drosophila* neurobiology

**DOI:** 10.1186/s12863-020-00895-7

**Published:** 2020-10-22

**Authors:** Anna A. Ogienko, Evgeniya N. Andreyeva, Evgeniya S. Omelina, Anastasiya L. Oshchepkova, Alexey V. Pindyurin

**Affiliations:** 1 Institute of Molecular and Cellular Biology, Siberian Branch of RAS, Novosibirsk, 630090 Russia; 2 Institute of Chemical Biology and Fundamental Medicine, Siberian Branch of RAS, Novosibirsk, 630090 Russia; 3 Novosibirsk State University, Novosibirsk, 630090 Russia

**Keywords:** *Drosophila*, Gal4/UAS, CNS, Neurons, Glia, Driver, *elav*-Gal4, *elav*^*C155*^, Gal4^*repo*^, 69B-Gal4

## Abstract

**Background:**

The *Drosophila* central nervous system (CNS) is a convenient model system for the study of the molecular mechanisms of conserved neurobiological processes. The manipulation of gene activity in specific cell types and subtypes of the *Drosophila* CNS is frequently achieved by employing the binary Gal4/UAS system. However, many Gal4 driver lines available from the Bloomington Drosophila Stock Center (BDSC) and commonly used in *Drosophila* neurobiology are still not well characterized. Among these are three lines with Gal4 driven by the *elav* promoter (BDSC #8760, #8765, and #458), one line with Gal4 driven by the *repo* promoter (BDSC #7415), and the 69B-Gal4 line (BDSC #1774). For most of these lines, the exact insertion sites of the transgenes and the detailed expression patterns of Gal4 are not known. This study is aimed at filling these gaps.

**Results:**

We have mapped the genomic location of the Gal4-bearing *P*-elements carried by the BDSC lines #8760, #8765, #458, #7415, and #1774. In addition, for each of these lines, we have analyzed the Gal4-driven GFP expression pattern in the third instar larval CNS and eye-antennal imaginal discs. Localizations of the endogenous Elav and Repo proteins were used as markers of neuronal and glial cells, respectively.

**Conclusions:**

We provide a mini-atlas of the spatial activity of Gal4 drivers that are widely used for the expression of UAS–target genes in the *Drosophila* CNS. The data will be helpful for planning experiments with these drivers and for the correct interpretation of the results.

## Background

Genetic and molecular studies in *Drosophila* can provide valuable insight into the pathogenesis of human diseases, due to the high conservation of key molecular mechanisms underlying biological processes in metazoans, and to the fact that about 77% human disease genes have orthologues in *Drosophila* [1]. Thus, targeted expression of mutant human disease genes in *Drosophila* can recapitulate relevant aspects of the pathology [2, 3]. Such expression is frequently achieved by employing the binary Gal4/UAS system, which allows the control of the activity of the gene of interest in a spatiotemporal-restricted manner [4]. The specificity of Gal4 expression allows cellular resolution that is particularly important for studies on the nervous system, in which there is a need to manipulate the activity of small sets of neurons or glia cells [5–8].

Anatomically, the *Drosophila* larval CNS comprises the brain and the ventral nerve cord (VNC), which are the equivalents of the vertebrate brain and spinal cord, respectively. The brain is composed of two main regions, the optic lobes located at the lateral surface of the brain hemispheres and the medially-located central brain [9]. Histologically, the CNS can be subdivided into the neuronal cell cortex, where all neuronal cell bodies reside, and the neuropil, where axons and dendrites project to form neural circuits [10]. Currently, a lot of different Gal4 drivers are available for targeted expression of reporter genes in distinct CNS cell types and subtypes. The choice of the proper driver for each experiment is crucial, since it can influence the results and their interpretation. However, for several widely-used Gal4 driver lines, the exact genomic location of the Gal4-bearing transgene and the precise pattern of Gal4 expression are still unknown. This information could be very useful (i) to learn whether the insertion of the Gal4 transgene disrupts the expression of an endogenous gene potentially relevant for the analysis undertaken and (ii) for combining the driver with other transgenes or mutations, so as to construct the desired genotype. Thus, we decided to identify the insertion sites of the transgenes and characterize Gal4 expression patterns in larval CNS for the following five commonly used CNS-specific driver lines available from BDSC: #8760, #8765, #458, #7415, and #1774.

In three lines, the Gal4 expression seems to be primarily under the control of the regulatory sequences of the *embryonic lethal, abnormal vision* (*elav*) gene, which is active in postmitotic neurons throughout development [11–13]. The lines #8760 and #8765 carry the P{Gal4-*elav*.L} element [14] somewhere on the third and second chromosomes, respectively. This *P*-element-based transposon contains a 3.5-kb genomic fragment including the *elav* promoter region [15] upstream of the Gal4 coding sequence. In line #458, an enhancer-trap Gal4 construct, P{GawB} [16], is inserted in the promoter of the *elav* gene (X:523350; here and afterwards, coordinates are from Release 6 of the *D. melanogaster* genome assembly [17]); this insertion is also known as the *elav*^*C155*^ allele [18]. Some differences between patterns/strength of activity of these drivers were previously described in epithelial tissues and embryonic CNS [19, 20]. Gal4 drivers from all the three lines are used to drive the expression of different genes and RNAi constructs in neuronal cells in CNS [21, 22].

In line #7415, the P{Gal4} element, presumably an enhancer-trap Gal4 construct, is inserted somewhere in the *reversed polarity* (*repo*) locus (3R:18236194..18239604; the insertion is referred to as Gal4^*repo*^) and the Gal4 expression pattern matches that of the endogenous Repo protein [23], which is specifically expressed in glia during development [24]. Contrary to the *elav*-Gal4 drivers, no activity of Gal4^*repo*^ was detected in larval epithelial tissues [19]; the highest level of *repo* expression in the larval CNS was observed in the optic lobes [24]. The Gal4^*repo*^ driver is commonly used to manipulate gene expression in glial cells in the CNS [25, 26].

Line #1774 carries the P{GawB} element inserted somewhere on the third chromosome. This insertion is known as 69B or 69B-Gal4 [16] and it drives expression of the reporter genes semi-ubiquitously. In third instar larvae, 69B-Gal4 activity was detected in the CNS, imaginal discs, ring gland, epidermis and testes [16, 27–29]. In the CNS, the expression of 69B-Gal4 seems to be restricted to non-glial cells [30].

Our previous experience indicates that transgenic *Drosophila* lines often carry more transposon constructs than commonly assumed, and that these insertions can potentially influence the experimental outcome [31]. In the present study, we identified the precise genomic locations of the Gal4 transgenes in lines #8760, #8765, #7415, and #1774, and confirmed the previous mapping of *elav*^*C155*^ in line #458. We also carefully characterized the expression pattern of a UAS-GFP reporter gene induced by the Gal4 drivers of these five lines in the CNS and eye-antennal imaginal discs of third instar larvae. We found that the enhancer-trap Gal4 drivers from lines #458 and #7415 elicit the expression patterns highly comparable to those of the endogenous Elav and Repo proteins, respectively. In contrast, the *elav*-Gal4 drivers from lines #8760 and #8765 match the Elav expression pattern only partially. In addition, our data support the view that the 69B-Gal4 driver is primarily active in non-glial cell types.

## Results

### Molecular characterization of the chromosomes bearing the Gal4 drivers

**Fig. 1:**
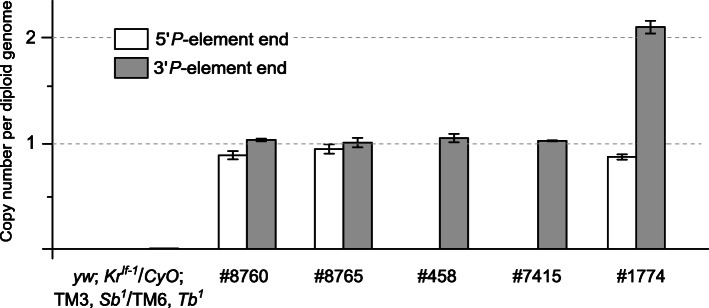
Copy numbers of *P*-element ends in different fly lines as measured by qPCR. One copy of *P*-element ends corresponds to a single transgene in the heterozygous flies. As expected, no *P*-element ends were detected in *yw*; *Kr*^*If-1*^/*CyO*; TM3, *Sb*^*1*^/TM6, *Tb*^*1*^ flies. In the BDSC lines #8760, #8765, #458, #7415, and #1774, all chromosomes except those carrying *P*-element insertions (based on the annotations of these lines available at BDSC website) were replaced by chromosomes from the line *yw*; *Kr*^*If-1*^/*CyO*; TM3, *Sb*^*1*^/TM6, *Tb*^*1*^ before qPCR analysis: #8760 (*yw*; *Kr*^*If-1*^/*CyO*; Chr3^#8760^/TM3, *Sb*^*1*^); #8765 (*yw*; Chr2^#8765^/*Kr*^*If-1*^; TM3, *Sb*^*1*^/TM6, *Tb*^*1*^); #458 (ChrX^#458^/*yw*; *Kr*^*If-1*^/*CyO*; TM3, *Sb*^*1*^/TM6, *Tb*^*1*^); #7415 (*yw*; *Kr*^*If-1*^/*CyO*; Chr3^#7415^/TM3, *Sb*^*1*^); #1774 (*yw*; *Kr*^*If-1*^/*CyO*; Chr3^#1774^/TM3, *Sb*^*1*^). In line #1774, two *P*-element transgenes were detected. The experiment was done in three replicates. Error bars represent standard errors

We first evaluated the number of *P*-element-based constructs harbored by the chromosomes Chr3^#8760^, Chr2^#8765^, ChrX^#458^, Chr3^#7415^, and Chr3^#1774^ bearing the Gal4 drivers in the five BDSC lines. To do that, we replaced all chromosomes (except the ones bearing the Gal4 constructs) in the original BDSC stocks with those from *yw*; *Kr*^*If-1*^/*CyO*; TM3, *Sb*^*1*^/TM6, *Tb*^*1*^ flies. Next, we performed qPCR analysis using genomic DNA from such fly lines with pairs of primers specific to the 5′ and 3′ *P*-element ends [31]. Notably, the minimal functional 5′ *P*-element end of 140 bp [32], present in the P{GawB} transposon and likely in P{Gal4} transposon, cannot be detected by these primers. qPCR indicates that there is only one *P*-element construct in each of the following chromosomes: Chr3^#8760^, Chr2^#8765^, ChrX^#458^, and Chr3^#7415^ (Fig. 1). However, Chr3^#1774^ seems to carry not only the P{GawB} construct but also another unexpected transposon possessing a long 5′ *P*-element end (Fig. 1).

**Fig. 2:**
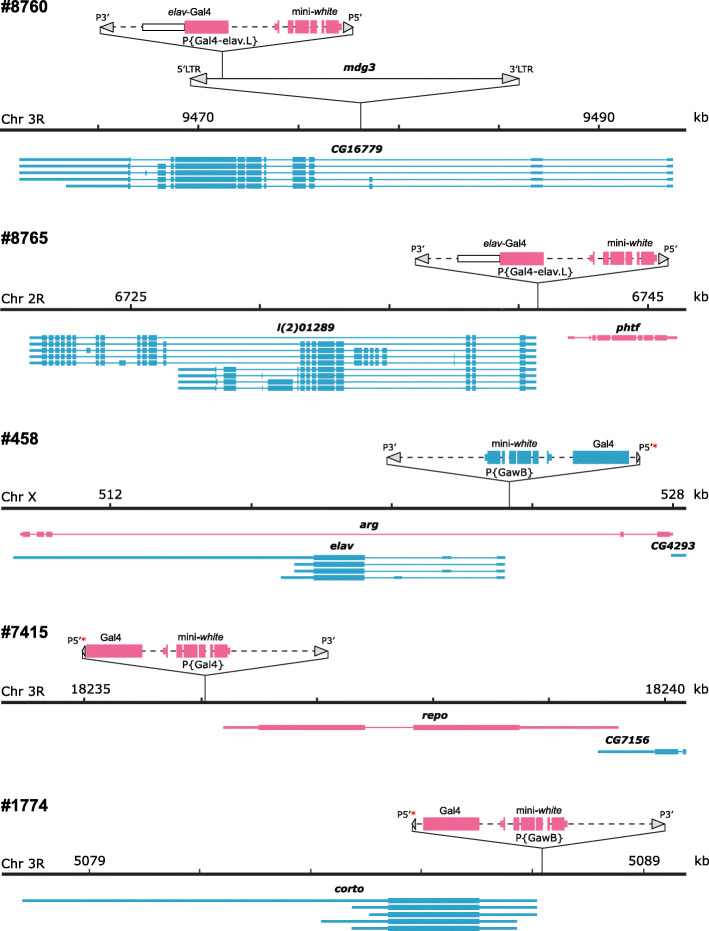
Insertion sites of *P*-element constructs found in the BDSC lines #8760, #8765, #458, #7415, and #1774. Thick horizontal black lines show segments of chromosomes flanking the insertion sites of *P*-element constructs. Genes on the forward and reverse strands are shown in magenta and blue, respectively. Coding sequences, UTRs and introns are represented as wide bars, narrow bars and lines, respectively. Grey triangles represent the 5′ and 3′ *P*-element ends, as well as the 5′ and 3′ long terminal repeats of the *mdg3* retrotransposon. Note that there are two types of 5′ *P*-element ends in the transposons: the “standard” ends of 585 bp and short ends of 140 bp, the latter are indicated by red asterisks. All *P*-element transgenes and the *mdg3* retrotransposon are not shown to scale. LTR, long terminal repeat

Next, we used an inverse PCR approach [33] to identify the insertion sites of the *P*-element constructs present in Chr3^#8760^, Chr2^#8765^, ChrX^#458^, Chr3^#7415^, and Chr3^#1774^ (Fig. 2). In Chr3^#8760^, the P{Gal4-*elav*.L} construct is inserted within an *mdg3* retrotransposon (at DNA sequence position 731–738, GenBank accession number X95908.1), which in turn is imbedded in the second/third intron of the *CG16779* gene (immediately after position 3R:9478115). In Chr2^#8765^, the insertion site of the P{Gal4-*elav*.L} construct is in the promoter region of the *l(2)01289* gene (2R:6740727–6740734). In ChrX^#458^, the P{GawB} construct is inserted in the promoter region of the *elav* gene (X:523343–523350), confirming the description of this line (https://flybase.org/reports/FBrf0211983). In Chr3^#7415^, the P{Gal4} construct is inserted in the *repo* gene promoter (3R:18236035–18236042). Notably, in ChrX^#458^ and Chr3^#7415^, the orientation of the Gal4 coding sequence matches that of the *elav* and *repo* genes. In Chr3^#1774^, the insertion site of P{GawB} element is in the promoter region of the *corto* gene (3R:5087163–5087170), but the orientation of the Gal4 coding sequence is opposite to the orientation of this gene. In addition, this chromosome carries an internally truncated *P*-element sequence of ~ 1.1 kb (corresponding to positions 3152–2931 and 1138–246 of GenBank accession number AB331393.1) inserted in the *distal antenna-related* (*danr*) gene promoter region (3R:25138044–25138051). The presence and location of all transposons was verified by PCR with primers specific to the *P*-element ends and genomic sequences flanking the insertion sites (Additional file 1: Table S1).

### Expression patterns of the Gal4 drivers

To reveal spatial activity of the Gal4 drivers carried by Chr3^#8760^, Chr2^#8765^, ChrX^#458^, Chr3^#7415^, and Chr3^#1774^ in the larval CNS and eye imaginal discs, we used a UAS-GFP reporter. Flies carrying the Gal4 drivers were crossed to UAS-GFP flies and the tissues dissected from the resulting progeny were immunostained with anti-Elav or anti-Repo antibodies and analyzed by confocal microscopy. GFP with a nuclear localization signal [34] was used because Elav and Repo are nuclear proteins [12, 35].

**Fig. 3:**
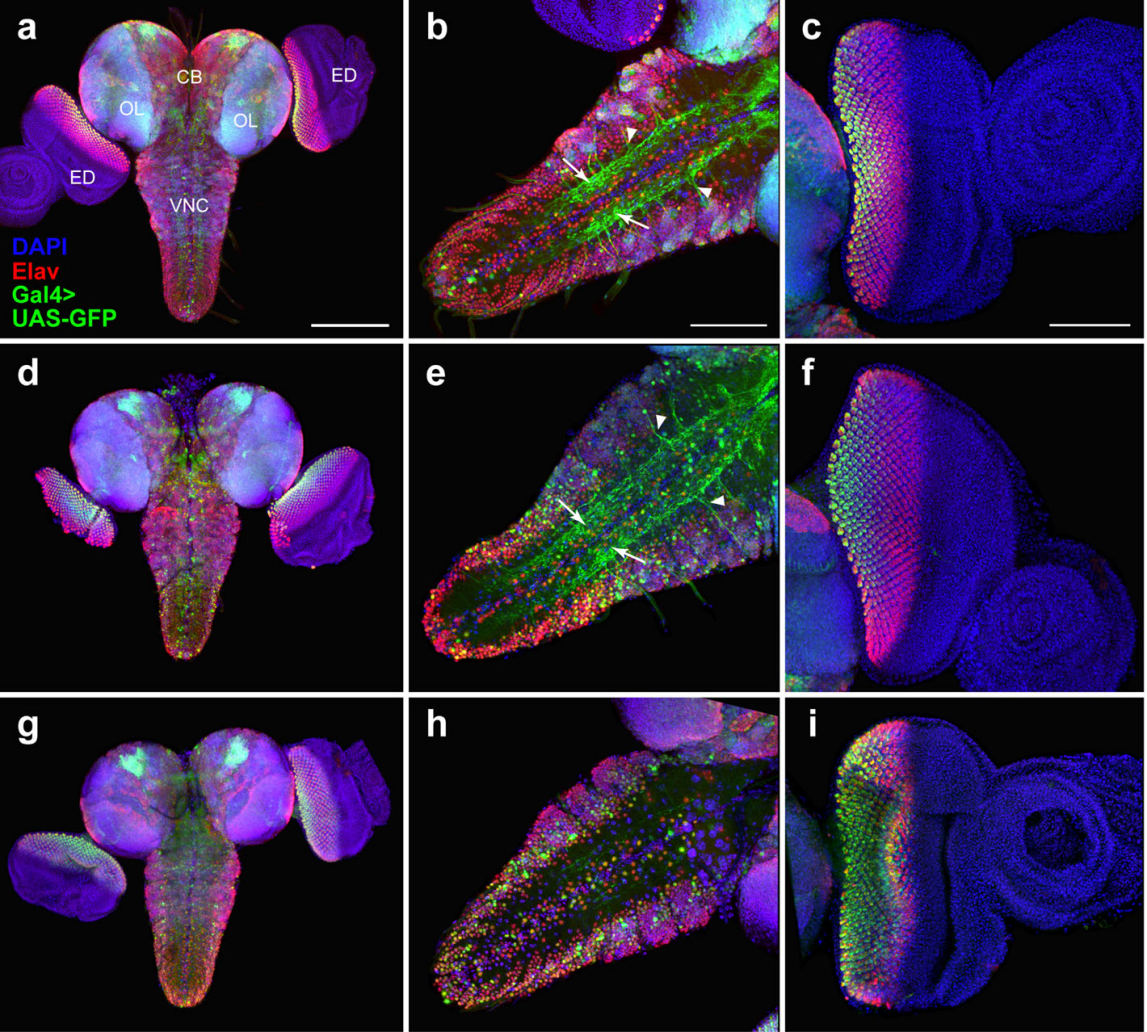
Comparison of GFP expression patterns elicited by the *elav*-Gal4 drivers of the BDSC lines #8760, #8765, and #458 with the Elav immunostaining pattern in the CNS and eye-antennal imaginal discs from third-instar larvae. Maximum intensity projections of confocal images of tissues stained with anti-Elav antibodies and showing GFP expression in the CNS and eye-antennal discs driven by Chr3^#8760^ (**a**-**c**), Chr3^#8765^ (**d**-**f**) or Chr3^#458^ (**g**-**i**). **a**, **d**, **g** The GFP and Elav patterns overlap only partially in the central brain and optic lobes. **b**, **e** Colocalization of the GFP and Elav signals in the VNC. Note the ectopic GFP expression in the neuropil (arrows) and in nerve roots (arrowheads). **c**, **f** In the eye imaginal discs, the pattern of the GFP expression is narrower compared to anti-Elav antibody staining. **h** Substantial colocalization of the GFP and Elav signals in the VNC. **i** In the eye imaginal discs, the patterns of GFP and Elav expression seem largely overlap. CB, central brain; OL, optic lobe; ED, eye imaginal disc; VNC, ventral nerve cord. Scale bars: **a**, **d**, **g**, 200 μm; **b**, **c**, **e**, **f**, **h**, **i**, 100 μm

In the larval CNS, the expression pattern of *elav*-Gal4 from lines #8760 and #8765 overlaps only partially with Elav immunostaining. The degree of colocalization of the GFP and Elav signals is higher in the VNC than in the central brain or the optic lobes (Fig. 3a-b, d-e; Additional files 2 and 3: Figures S1 and S2). We also observed ectopic GFP expression in the neuropil and nerve roots (Fig. 3b, e). In the eye imaginal discs, the endogenous Elav protein is expressed in photoreceptor neurons and therefore marks the developing eye area posterior to the morphogenetic furrow [36]. We found that the *elav*-driven expression of Gal4 in lines #8760 and #8765 does not fully reproduce the pattern of Elav immunostaining, as the GFP signals occupy a narrower area compared to the Elav signal (Fig. 3c, f; Additional file 4: Figure S3).

Also, in the CNS from line #458 bearing the *elav*^*C155*^ allele, the overlapping of the GFP and Elav signals is only partial. Signal colocalization is evident in the VNC but there is no ectopic GFP expression in the neuropil (Fig. 3g, h; Additional file 5: Figure S4). In the eye imaginal discs of *elav*^*C155*^ larvae, the GFP expression pattern is almost completely overlapping the Elav immunostaining (Fig. 3i; Additional files 4 and 5: Figures S3 and S4).

**Fig. 4:**
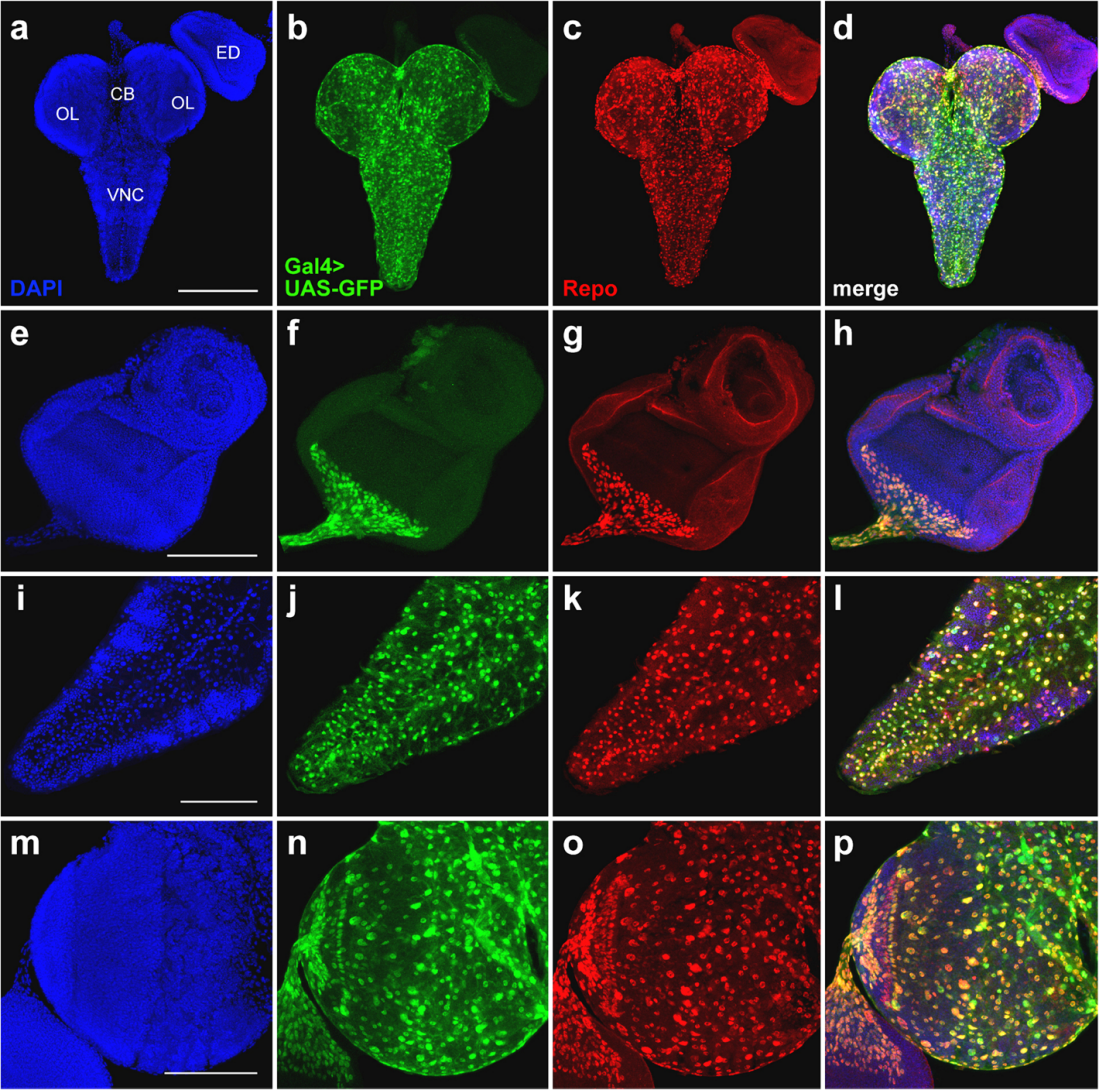
Comparison of GFP expression patterns controlled by the Gal4^*repo*^ driver from the BDSC line #7415 with the Repo expression pattern in the CNS and eye-antennal imaginal discs from third-instar larvae. Maximum intensity projections of confocal images of tissues stained with anti-Repo antibodies are shown. **a**-**d** Substantial overlap between the GFP and Repo signals in the CNS and eye imaginal discs. **e**-**h** Perfect colocalizations of the GFP and Repo signals in the eye imaginal disc and optic stalk. **i**-**l** Colocalization of the GFP and Repo expression patterns in the VNC. **m**-**p** Overlapping of the GFP and Repo signals in the central brain and optic lobes; note that the GFP expression pattern seems to be broader than the Repo immunostaining pattern. CB, central brain; OL, optic lobe; ED, eye imaginal disc; VNC, ventral nerve cord. Scale bars: **a**-**d**, 200 μm; **e**-**p**, 100 μm

In the optic lobes and eye imaginal discs, the GFP expression pattern directed by the Gal4^*repo*^driver of line #7415 is almost coincidental with the localization of the Repo protein (Fig. 4; Additional file 6: Figure S5). However, in both the VNC and central brain, the GFP and Repo localization patterns are slightly different (Fig. 4i-l; Additional file 6: Figure S5).

**Fig. 5:**
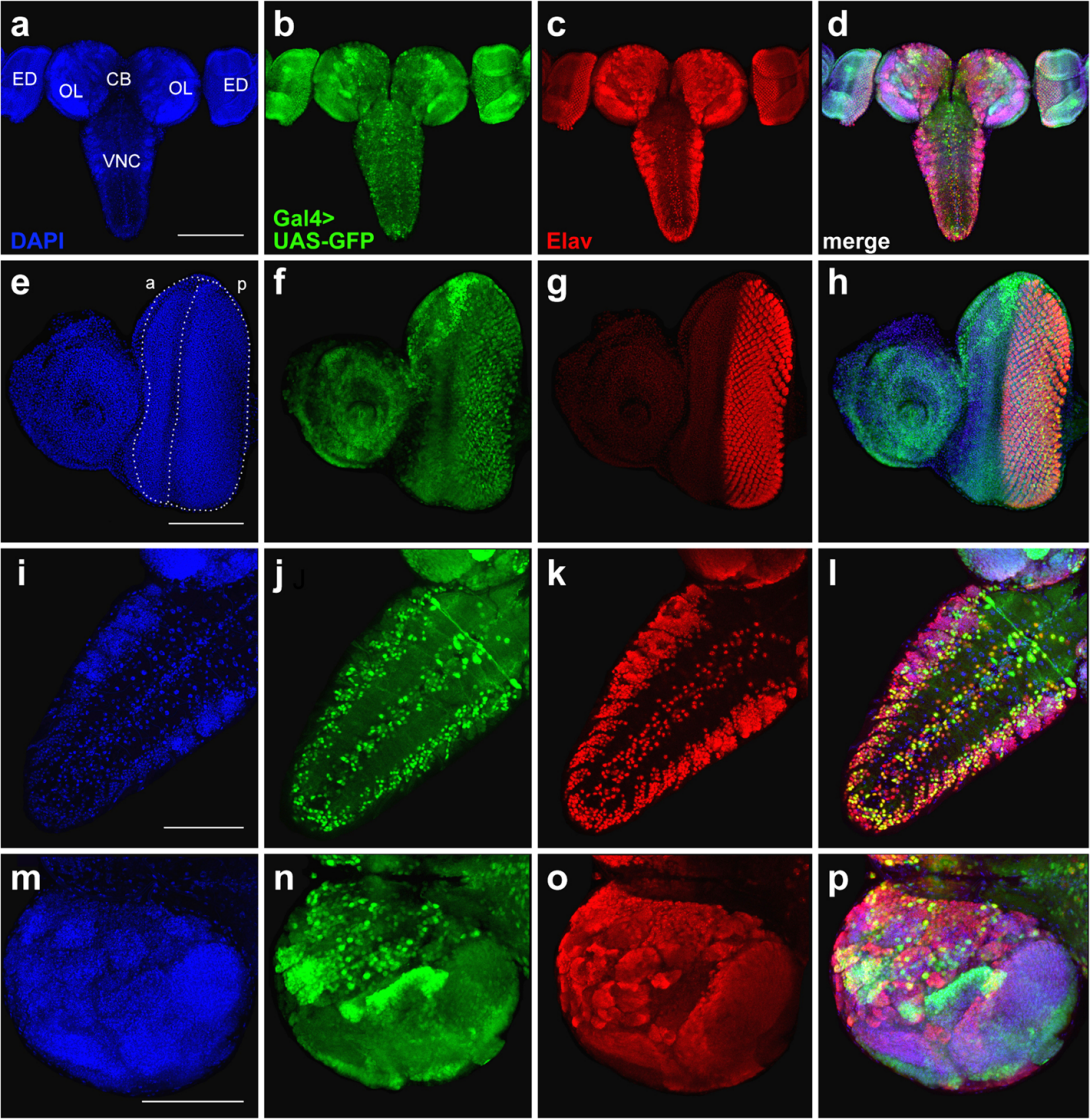
Comparison of GFP expression patterns elicited by the 69B-Gal4 driver from the BDSC line #1774 with the Elav immunostaining pattern in the CNS and eye-antennal imaginal discs from third-instar larvae. Maximum intensity projections of confocal images of tissues stained with anti-Elav antibodies are shown. **a**-**d** Overlapping of the GFP expression pattern and the Elav localization in the CNS and eye imaginal discs. **e**-**h** In the eye-antennal disc, the Elav staining is largely coincident with the GFP signal, but the GFP expression is much broader than that of the Elav protein and includes the anterior area of the eye disc and the antennal disc. **i**-**l** Partial colocalization of the GFP and Elav expression patterns in the VNC. **m**-**p** Partial overlap between the GFP and Elav signals in the central brain and optic lobes. Note that GFP is expressed only in subset of Elav-positive cells in the VNC. CB, central brain; OL, optic lobe; ED, eye imaginal disc; a, anterior part of the disc; p, posterior part of the disc; VNC, ventral nerve cord. Scale bars: **a**-**d**, 200 μm; **e**-**p**, 100 μm

**Fig. 6:**
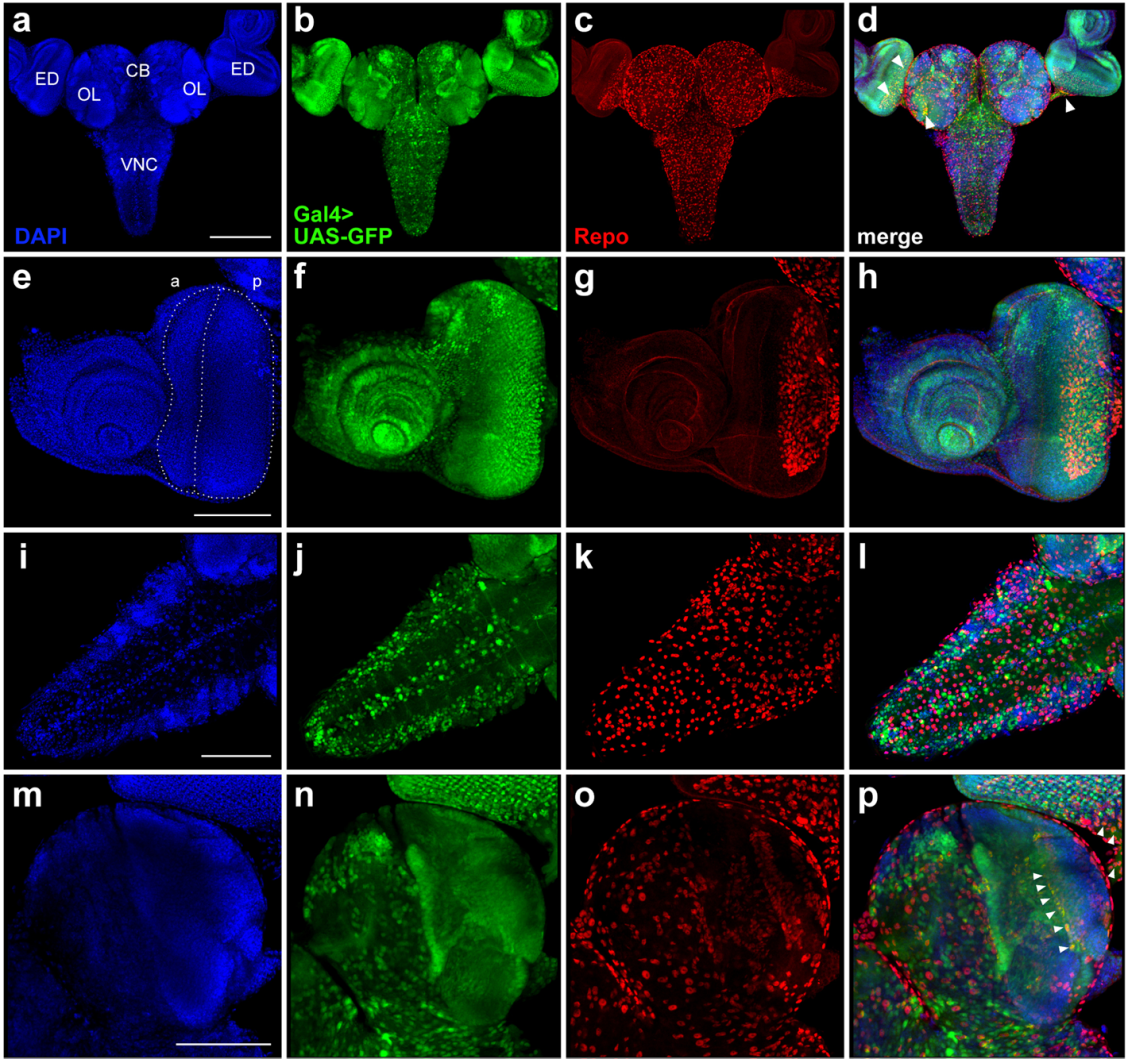
Comparison of GFP expression patterns elicited by 69B-Gal4 driver from the BDSC line #1774 with the Repo expression pattern in the CNS and eye-antennal imaginal discs from third-instar larvae. Maximum intensity projections of confocal images of tissues stained with anti-Repo antibodies are shown. **a**-**d** In the CNS and eye-antennal imaginal discs, the GFP expression pattern overlaps with that of Repo just in individual groups of cells (arrowheads). **e**-**h** In the eye-antennal imaginal discs, the broad GFP expression pattern overlaps with the Repo protein expression pattern only in the optic stalk and in the posterior part of the eye disc. **i-l** The GFP and Repo expression patterns in the VNC are not overlapping. **m-p** The GFP and Repo signals minimally overlap in the central brain and optic lobes; cells showing both signals are indicated by arrowheads. CB, central brain; OL, optic lobe; ED, eye imaginal disc; a, anterior part of the disc; p, posterior part of the disc; VNC, ventral nerve cord. Scale bars: **a**-**d**, 200 μm; **e**-**p**, 100 μm

To characterize the expression pattern of the 69B-Gal4 driver of line #1774, we compared the GFP localization elicited by this driver with the distribution of the Elav and Repo proteins in larval CNS and eye-antennal imaginal discs. The GFP and Elav signals substantially colocalize in the VNC and eye imaginal discs, but colocalization is limited in the central brain and the optic lobes. In addition, the 69B-Gal4 driver is heavily expressed in the antennal disc, where Elav is not present (Fig. 5; Additional file 7: Figure S6). A comparison between the 69B-Gal4 and Repo expression patterns revealed that individual Repo-positive cells in the central brain, optic lobes and optic stalk also express GFP. In the eye-antennal disc, Repo is expressed only in the posterior part of the eye disc, where it partially overlaps with the GFP signal (Fig. 6; Additional file 8: Figure S7). Altogether, these results suggest that the 69B-Gal4 driver is mainly active in neuronal cells, although it is also expressed in other cell types, including imaginal disc cells and small subsets of glial cells.

## Discussion

The accessibility of the CNS of *Drosophila* third instar larvae and its similarities to the developing mammalian nervous system make it a very attractive model system. Here, we have characterized five transgenic fly lines carrying commonly used Gal4 drivers for neurobiological studies. Specifically, we identified the genomic insertion sites of the Gal4 transgenes and visualized the Gal4 activity patterns using a UAS-GFP reporter. These patterns were compared to the localization of the Elav and Repo proteins that are believed to be specific for neuronal and glial cell types, respectively.

In lines #8760 and #8765, the functions of the *CG16779* and *l(2)01289* genes might be compromised by the insertion of the *elav*-Gal4 construct. Information on these genes and their expression profiles is currently scarce. However, both genes were found to regulate *Drosophila* lifespan [37, 38], suggesting some caution in the interpretation the longevity experiments, in which the *elav*-Gal4 drivers were used [39–41]. In addition, the *CG16779* gene is predicted to encode a factor involved in regulation of Choline acetyltransferase (ChAT), which is responsible for the synthesis of the neurotransmitter acetylcholine [42]. In line #1774, *P*-element insertions resulted in mutations in the *corto* and *danr* genes. The *corto* gene encodes an Enhancer of Trithorax and Polycomb (ETP) protein, which might be involved in the maintenance of gene expression patterns throughout development [43]. In the CNS of third instar larvae, the expression of *corto* was detected in the optic lobes and the VNC [44]. The *danr* gene encodes a putative transcription factor, which is involved in the retinal determination network during eye development [45, 46]. Lastly, it is not clear whether the insertions of the enhancer-trap Gal4 constructs in the lines #458 and #7415 somehow affect the expression of the *elav* and *repo* genes, respectively. Nevertheless, all these Gal4 driver lines, with the exception of #8765, are homozygous or hemizygous viable.

The partial overlap of Gal4 expression from lines #8760 and #8765 with the localization of the Elav protein in the CNS and eye imaginal discs, as well as ectopic Gal4 expression in the neuropil, are not so surprising. This finding is probably related to the absence of important regulatory sequences controlling the expression pattern of the endogenous *elav* gene within the P{Gal4-*elav*.L} transposon. Particularly, it was recently reported that the *elav* gene is ubiquitously transcribed and post-transcriptionally repressed in non-neural tissues via its 3′ UTR sequence [47], which is absent in the P{Gal4-*elav*.L} construct. At the same time, the influence of the genomic location on the activity of the *elav*-Gal4 construct in lines #8760 and #8765 seems to be negligible, as both drivers elicit almost identical Gal4 expression patterns.

More interesting are the observations made for the enhancer-trap Gal4 drivers *elav*^*C155*^ and Gal4^*repo*^ that were expected to mimic the expression patterns of the genes, in which they are integrated. These drivers do indeed show substantial, but not complete, overlap between the Gal4 expression and Elav and Repo immunostaining, respectively. This indicates that some regulatory signals, that normally control the activity of the *elav* and *repo* genes, became inactive or improperly functional upon the insertion of the transgenes. The presence of the *P*-transposase promoter and the mini-*white* reporter gene in the P{GawB} and P{Gal4} elements might be responsible for the phenomenon.

The unique expression pattern of 69B-Gal4 driver is most likely the result of interactions of the *P*-transposase promoter located upstream of the Gal4 coding sequence in the P{GawB} construct with the enhancers and some other regulatory elements from the *corto* locus.

## Conclusions

Most of the currently available Gal4 drivers used in *Drosophila* neurobiology elicit expression patterns that only partially overlap those of the endogenous proteins. Transgenes encoding Gal4 under the control of promoters of particular genes may lack important regulatory elements that are normally located either outside the genes or in their exon-intron sequences. In addition, expression of such Gal4 transgenes may depend on their genomic location. The enhancer-trap Gal4 constructs, although showing expression patterns similar to those of the genes in which they are integrated, also seem to lack some regulatory expression signals. Thus, a perfect copying of the endogenous protein expression patterns by Gal4 drivers appears to be a very complex task. To accomplish this task, it would be necessary to identify all factors affecting the activity of the gene studied. Until this is achieved, all available information on the expression patterns of Gal4 drivers, should be taken into account in both experimental design and the interpretation of the results.

## Methods

### Fly stocks

Flies were raised and crossed on standard cornmeal agar media at 25 °C. The fly stocks carrying driver constructs used in this study were obtained from the BDSC (Bloomington, IN, USA; https://bdsc.indiana.edu/): #8760 (w^*^; P{*w*^*+mC*^ = Gal4-*elav*.L}3); #8765 (P{*w*^*+mC*^ = Gal4-*elav*.L}2/*CyO*); #458 (P{*w*^*+mW.hs*^ = GawB}*elav*^*C155*^); #7415 (*w*^*1118*^; P{*w*^+m*^ = Gal4}*repo*/TM3, *Sb*^*1*^); #1774 (w^*^; P{*w*^*+mW.hs*^ = GawB}69B); #4775 (*w*^*1118*^; P{*w*^*+mC *^= UAS-GFP.nls}14). Line *yw*; *Kr*^*If-1*^/*CyO*; TM3, *Sb*^*1*^/TM6, *Tb*^*1*^ was provided by the “Molecular and Cellular Biology” core facility of the IMCB SB RAS.

### Genomic DNA extraction and determination of *P*-element transgene copy number

Genomic DNA was isolated from 30 to 50 flies according to the protocol reported earlier [48]. For detection of *P*-element-based transgene copy number by qPCR, we used the reference plasmid pP5′-Vps36-759bp-P3′ described previously [31]. The following three primer pairs were used: Vps36-realtime-F and Vps36-realtime-R specific for the *Vps36* gene, qP5-F1 and qP5-R1 for the 5′ *P*-element end, and qP3-F1 and qP3-R1 for the 3′ *P*-element end (for primer sequences, see [31]). Importantly, primers qP5-F1 and qP5-R1 can detect only the “standard” 5′ *P*-element end of 585 bp in length, but not the minimal functional 5′ *P*-element end of 140 bp in length that is present in enhancer-trap Gal4 transposons. qPCR was performed with 100 ng of genomic DNA or 5 pg of the reference plasmid pP5′-Vps36-759bp-P3′, 400 nM of each primer in a 25-μl reaction mixture using the HS-qPCR SYBR Blue Master Mix (Biolabmix), and the CFX96 Touch Real-Time PCR Detection System (Bio-Rad) under the following conditions: incubation at 95 °C for 5 min, followed by 39 cycles of 95 °C for 15 s, 60 °C for 30 s, and 72 °C for 30 s. Data analysis was performed using CFX Manager™ Software v3.0 (Bio-Rad). The 5′ and 3′ *P*-element copy numbers per diploid genome were calculated according to [31].

### Mapping *P*-element transgene insertion sites

Mapping of *P*-element transgene insertion sites was done by inverse-PCR [33] according to “Inverse PCR & Cycle Sequencing of P Element Insertions for STS Generation” protocol of E.J. Rehm (Berkeley Drosophila Genome Project; www.fruitfly.org/about/methods/inverse.pcr.html) with the following modifications. One microgram of genomic DNA was digested with HhaI (New England Biolabs) or Kzo9I (SibEnzyme) or MspI (SibEnzyme) or SalI (New England Biolabs) restriction enzyme for 6 h at 37 °C in a volume of 50 μl. Restriction fragments were circularized by incubation with T4 DNA ligase (Thermo Scientific) and then purified using Microcon Ultracel YM-30 centrifugal filter (Millipore), followed by elution of DNA in 40 μl of nuclease-free water. Five microliters of each sample was used as a template for PCR amplification of fragments containing the *P*-element end (5′ or 3′) and flanking genomic DNA. PCR reactions were performed using Phusion® High-Fidelity DNA Polymerase (New England Biolabs) and the following two primer pairs: Plac1 and Plac4 specific for the 5′ *P*-element end of 585 bp (but not of 140 bp) in length, and Pry1 and Pry2 specific for the 3′ *P*-element end (for primer sequences, see Additional file 1: Table S1). The gel-purified PCR products were cloned into pBluescript II SK+ vector (Promega) for subsequent sequencing using universal primers pBS-F1 (5′-cagggttttcccagtcacgac-3′) and pBS-R1 (5′-ggctttacactttatgcttcc-3′).

### PCR genotyping

PCR was performed using Hot-Start Taq DNA polymerase (Biolabmix) according to the manufacturer’s recommendations. Details of primer pairs used for genotyping all *P*-element transposons are provided in Additional file 1: Table S1. The PCR products were analyzed on 1% agarose gel along with an appropriate DNA marker.

### Immunofluorescence staining and confocal microscopy

The CNS and attached eye-antennal imaginal discs were dissected from third instar larvae. For direct GFP detection (without GFP immunostaining) and antibody staining, tissues were fixed in phosphate-buffered saline (PBS) containing 4% formaldehyde (Merck), and then washed 3 times (5 min each) with 0.5% Triton X-100 in PBS. The primary monoclonal rat anti-Elav (DSHB #7E8A10) and monoclonal mouse anti-Repo (DSHB #8D12) antibodies were used at concentration 1 μg/ml. They were detected by goat anti-rat IgG antibodies conjugated to AlexaFluor568 (1:800; Invitrogen #A-11077) and by goat anti-mouse IgG antibodies conjugated to AlexaFluor568 (1:800; Invitrogen #A-11031). Finally, tissues were stained with 0.4 μg/ml DAPI dissolved in PBS. All samples were imaged at the same settings using confocal microscope LSM 710 (Carl Zeiss) with 10×/0.45 plan-apo and 20×/0.8 plan-apo lenses. Optical sections were combined using the LSM Image Browser version 4.2 software (Carl Zeiss).

## Supplementary information

**Additional file 1: Table S1.** Primers used for PCR verification of transposon insertion sites and details of PCR products.

**Additional file 2: Figure S1.** Representative series of z-stack confocal images of the CNS and eye-antennal imaginal discs from a third-instar larva expressing GFP under the control of the *elav*-Gal4 driver from the BDSC line #8760. The tissues are stained with anti-Elav antibodies.

**Additional file 3: Figure S2.** Representative series of z-stack confocal images of the CNS and eye-antennal imaginal discs from a third-instar larva expressing GFP under the control of the *elav*-Gal4 driver from the BDSC line #8765. The tissues are stained with anti-Elav antibodies.

**Additional file 4: Figure S3.** Comparison of GFP expression patterns elicited by the Gal4 drivers from the BDSC lines #8760, #8765, and #458 with the Elav immunostaining pattern in the eye-antennal imaginal discs from third-instar larvae. CB, central brain; OL, optic lobe. Scale bar: 100 μm.

**Additional file 5: Figure S4.** Representative series of z-stack confocal images of the CNS and eye-antennal imaginal discs from a third-instar larva expressing GFP under the control of the *elav*^*C155*^ driver from the BDSC line #458. The tissues are stained with anti-Elav antibodies.

**Additional file 6: Figure S5.** Representative series of z-stack confocal images of the CNS and eye-antennal imaginal discs from a third-instar larva expressing GFP under the control of the Gal4^*repo*^ driver from the BDSC line #7415. The tissues are stained with anti-Repo antibodies.

**Additional file 7: Figure S6.** Representative series of z-stack confocal images of the CNS and eye-antennal imaginal discs from a third-instar larva expressing GFP under the control of the 69B-Gal4 driver from the BDSC line #1774. The tissues are stained with anti-Elav antibodies.

**Additional file 8: Figure S7.** Representative series of z-stack confocal images of the CNS and eye-antennal imaginal discs from a third-instar larva expressing GFP under the control of the 69B-Gal4 driver from the BDSC line #1774. The tissues are stained with anti-Repo antibodies.

## Data Availability

All materials are available upon request.

## Abbreviations

BDSC: Bloomington Drosophila Stock Center
CB: Central brain
CNS: Central nervous system
DSHB: Developmental Studies Hybridoma Bank
Elav: Embryonic lethal, abnormal vision
GFP: Green fluorescent protein
LTR: Long terminal repeats
PBS: Phosphate-buffered saline
Repo: Reverse polarity
VNC: Ventral nerve code
UAS: Upstream activation sequence
UTR: Untranslated region
