# Supplementary material for: Molecular and cytological analysis of widely-used Gal4 driver lines for Drosophila neurobiology
Source: BMC Genet. 2020 Oct 22;21(Suppl 1):96. doi: 10.1186/s12863-020-00895-7 (PMC7583314; doi:10.1186/s12863-020-00895-7)
Supplement: Supplementary file 1 — Additional file 1: Table S1. Primers used for PCR verification of transposon insertion sites and details of PCR products. [file 12863_2020_895_MOESM1_ESM.pdf]

Table S1. Primers used for PCR verification of transposon insertion sites and details of PCR products.

Transgene	Primer name	Primer sequence (5′→>3′)	PCR products*	
			from the corresponding transgenic line genomic DNA	from wild-type genomic DNA
elav-Gal4 driver construct in Chr3[#8760]	BL8760-F<br>Pry2	cattgcagacagacttgatggtcag<br>cttgccgacgggaccaccttatgttatt	1388 bp	-
	Plac1<br>BL8760-R	cacccaaggctctgctcccacaat<br>ggaaattttgtagcggtatgc	761 bp	-
	BL8760-F<br>BL8760-R	cattgcagacagacttgatggtcag<br>ggaaattttgtagcggtatgc	-	-
	BL8760-F<br>Gal4-R	cattgcagacagacttgatggtcag<br>atgatgatgtcgcacttattctatgc	~5300 bp	-
	Gal4-F<br>mWhite-R	caaccaattgcctcctctaacg<br>cgaattaatagctcctgatcctc	~1500 bp	-
elav-Gal4 driver construct in Chr2[#8765]	BL8765-F<br>Pry2	tgcaaagacgatttctcacagc<br>cttgccgacgggaccaccttatgttatt	1092 bp	-
	Plac1<br>BL8765-R	cacccaaggctctgctcccacaat<br>acaatgtcacgttatgtctcttagc	635 bp	-
	BL8765-F<br>BL8765-R	tgcaaagacgatttctcacagc<br>acaatgtcacgttatgtctcttagc	1427 bp	1427 bp
	BL8765-F<br>Gal4-R	tgcaaagacgatttctcacagc<br>atgatgatgtcgcacttattctatgc	~5000 bp	-
	Gal4-F<br>mWhite-R	caaccaattgcctcctctaacg<br>cgaattaatagctcctgatcctc	~1500 bp	-

Continued on the next page

Transgene	Primer name	Primer sequence (5′→>3′)	PCR products* from the corresponding transgenic line genomic DNA	PCR products* from wild-type genomic DNA
elav^{C155} driver construct in ChrX$^{\#458}$	BL458-F<br>Pry2	ggatactcttacatttcgctcg<br>cttgccgacgggaccaccttatgttatt	413 bp	-
	P5short-R<br>BL458-R	ctatcgacgggaccaccttatg<br>tggcgatatatggcatacagtatg	1059 bp	-
	BL458-F<br>BL458-R	ggatactcttacatttcgctcg<br>tggcgatatatggcatacagtatg	-	1393 bp
	Gal4-R<br>BL458-R	atgatgatgtcgcacttattctatgc<br>tggcgatatatggcatacagtatg	1641 bp	-
	Gal4-F<br>mWhite-R	caaccaattgcctcctctaacg<br>cgaattaatagctcctgatcctc	1419 bp	-
Gal4^{repo} driver construct in Chr3$^{\#7415}$	BL7415-F<br>P5short-R	taggataacaataagatcagcgtgc<br>ctatcgacgggaccaccttatg	351 bp	-
	Pry2<br>BL7415-R	cttgccgacgggaccaccttatgttatt<br>tcgtcttcttcttcttgatgcc	1420 bp	-
	BL7415-F<br>BL7415-R	taggataacaataagatcagcgtgc<br>tcgtcttcttcttcttgatgcc	-	1692 bp
	BL7415-F<br>Gal4-R	taggataacaataagatcagcgtgc<br>atgatgatgtcgcacttattctatgc	933 bp	-
	Gal4-F<br>mWhite-R	caaccaattgcctcctctaacg<br>cgaattaatagctcctgatcctc	1419 bp	-

Continued on the next page

Transgene	Primer name	Primer sequence (5′→>3′)	PCR products* from the corresponding transgenic line genomic DNA	PCR products* from wild-type genomic DNA
69B-Gal4 driver construct in Chr3[#1774]	BL1774-F<br>P5short-R	gcttttgctctcgtttcggtaa<br>ctatcgacgggaccaccttatg	1157 bp	-
	Pry2<br>BL1774-R	cttgccgacgggaccaccttatgttatt<br>caccataaaagtcggcataaagc	1515 bp	-
	BL1774-F<br>BL1774-R	gcttttgctctcgtttcggtaa<br>caccataaaagtcggcataaagc	-	2593 bp
	BL1774-F<br>Gal4-R	gcttttgctctcgtttcggtaa<br>atgatgatgtcgcacttattctatgc	1739 bp	-
	Gal4-F<br>mWhite-R	caaccaattgcctcctctaacg<br>cgaattaatagctcctgatcctc	1419 bp	-
the internally truncated *P*-element in Chr3[#1774]	danr-F<br>Plac4	ctttgctccgattggtcgag<br>actgtgcgttaggtcctgttcattgtt	707 bp	-
	Pry1<br>danr-R	ccttagcatgtccgtggggtttgaat<br>cgaactcgcagaaggatgtg	1069 bp	-
	danr-F<br>danr-R	ctttgctccgattggtcgag<br>cgaactcgcagaaggatgtg	1289 bp	165 bp

* PCR products were obtained using Hot-Start Taq DNA polymerase (Biolabmix) with the following program: 95°C for 30 s, followed by 35 cycles of 95°C for 30 s, 58 or 60°C for 60 s, and 72°C for *X* min (*X* was dependent on amplicon length, but it was never longer than 5 min), with a final cycle at 72°C for 5 min.
