# Supplementary figures and images for: Molecular and cytological analysis of widely-used Gal4 driver lines for Drosophila neurobiology
Source: BMC Genet. 2020 Oct 22;21(Suppl 1):96. doi: 10.1186/s12863-020-00895-7 (PMC7583314; doi:10.1186/s12863-020-00895-7)

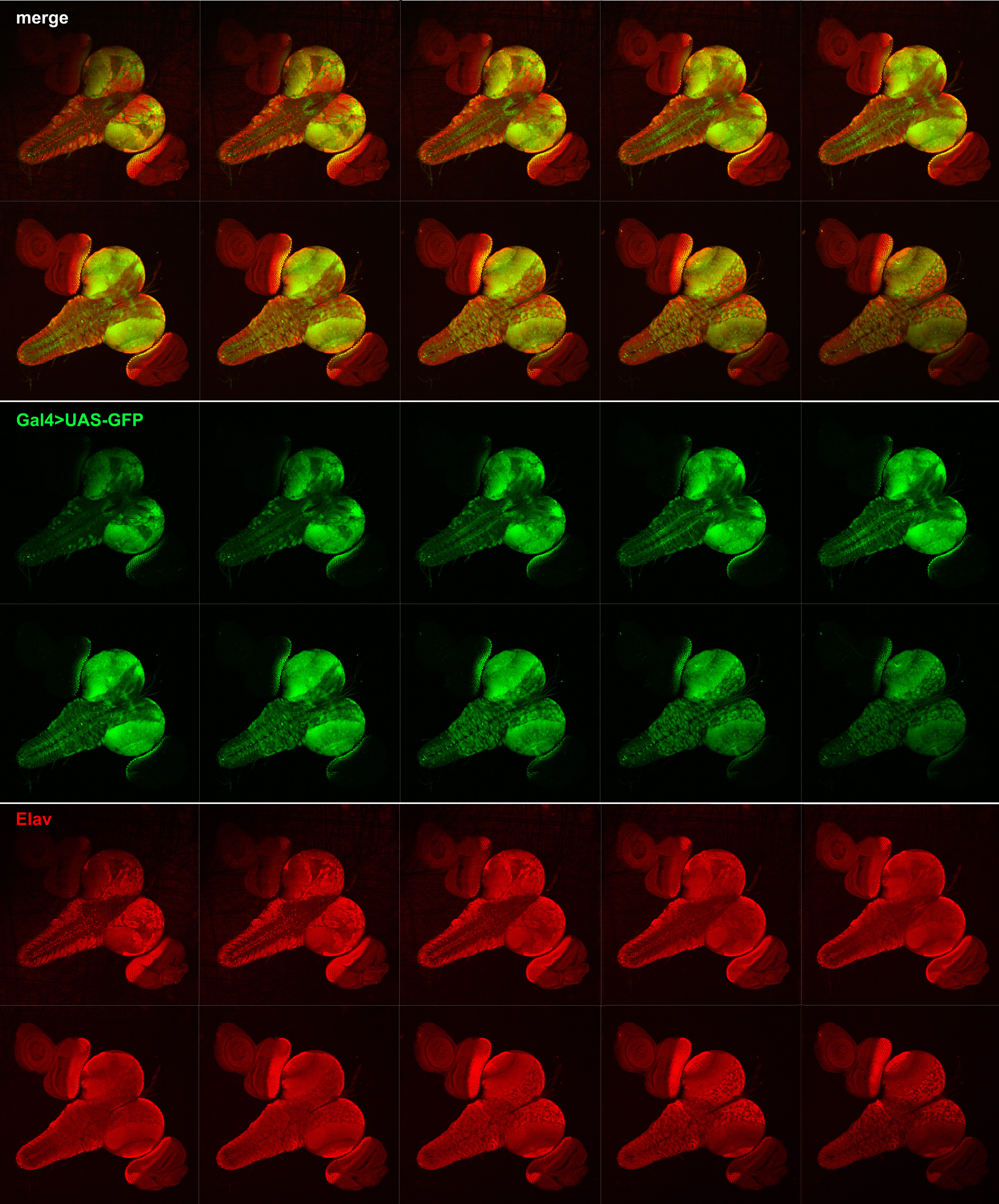

Supplement: Supplementary file 2 — Additional file 2: Figure S1. Representative series of z-stack confocal images of the CNS and eye-antennal imaginal discs from a third-instar larva expressing GFP under the control of the elav-Gal4 driver from the BDSC line #8760. The tissues are stained with anti-Elav antibodies. [file 12863_2020_895_MOESM2_ESM.jpg]

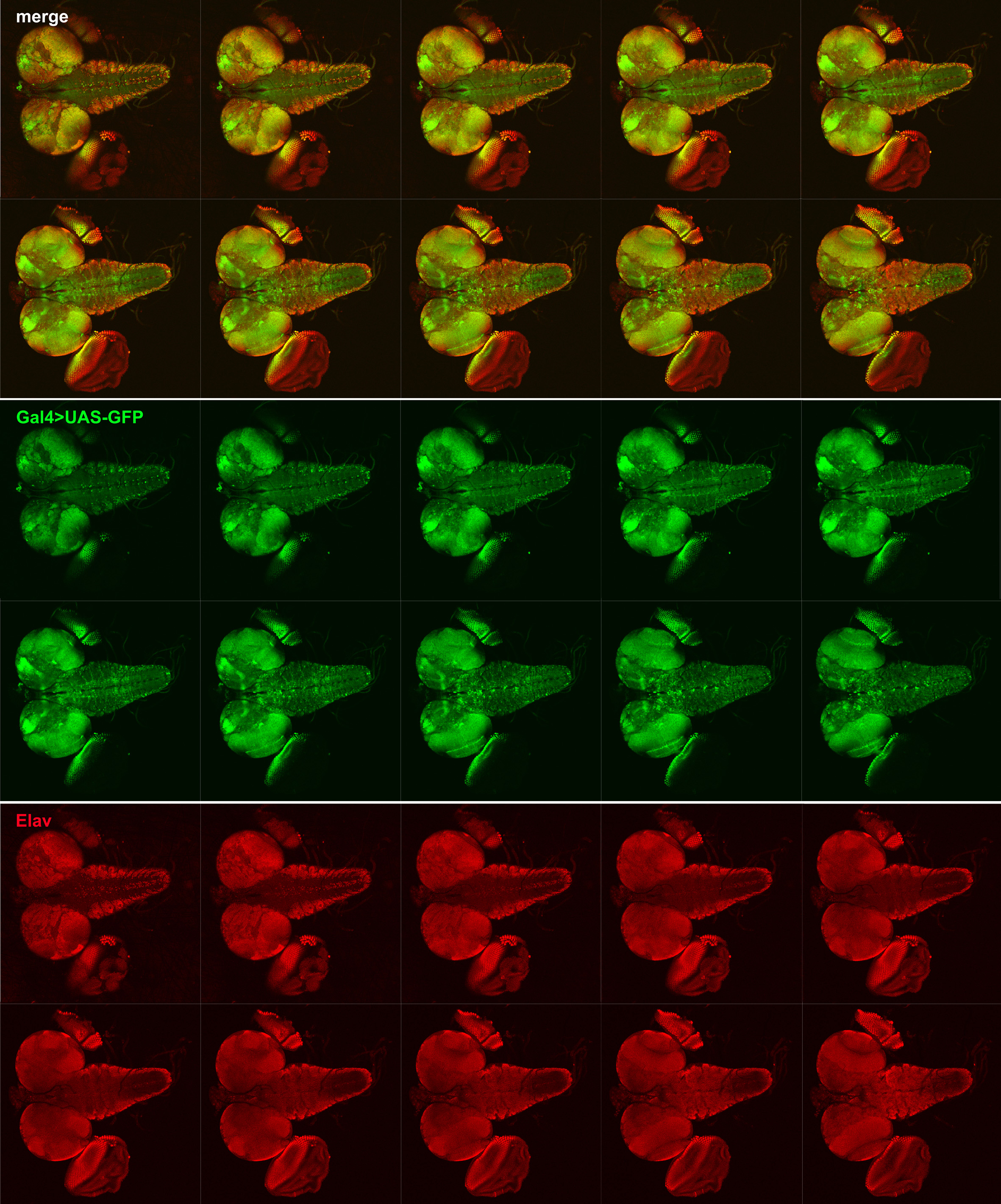

Supplement: Supplementary file 3 — Additional file 3: Figure S2. Representative series of z-stack confocal images of the CNS and eye-antennal imaginal discs from a third-instar larva expressing GFP under the control of the elav-Gal4 driver from the BDSC line #8765. The tissues are stained with anti-Elav antibodies. [file 12863_2020_895_MOESM3_ESM.jpg]

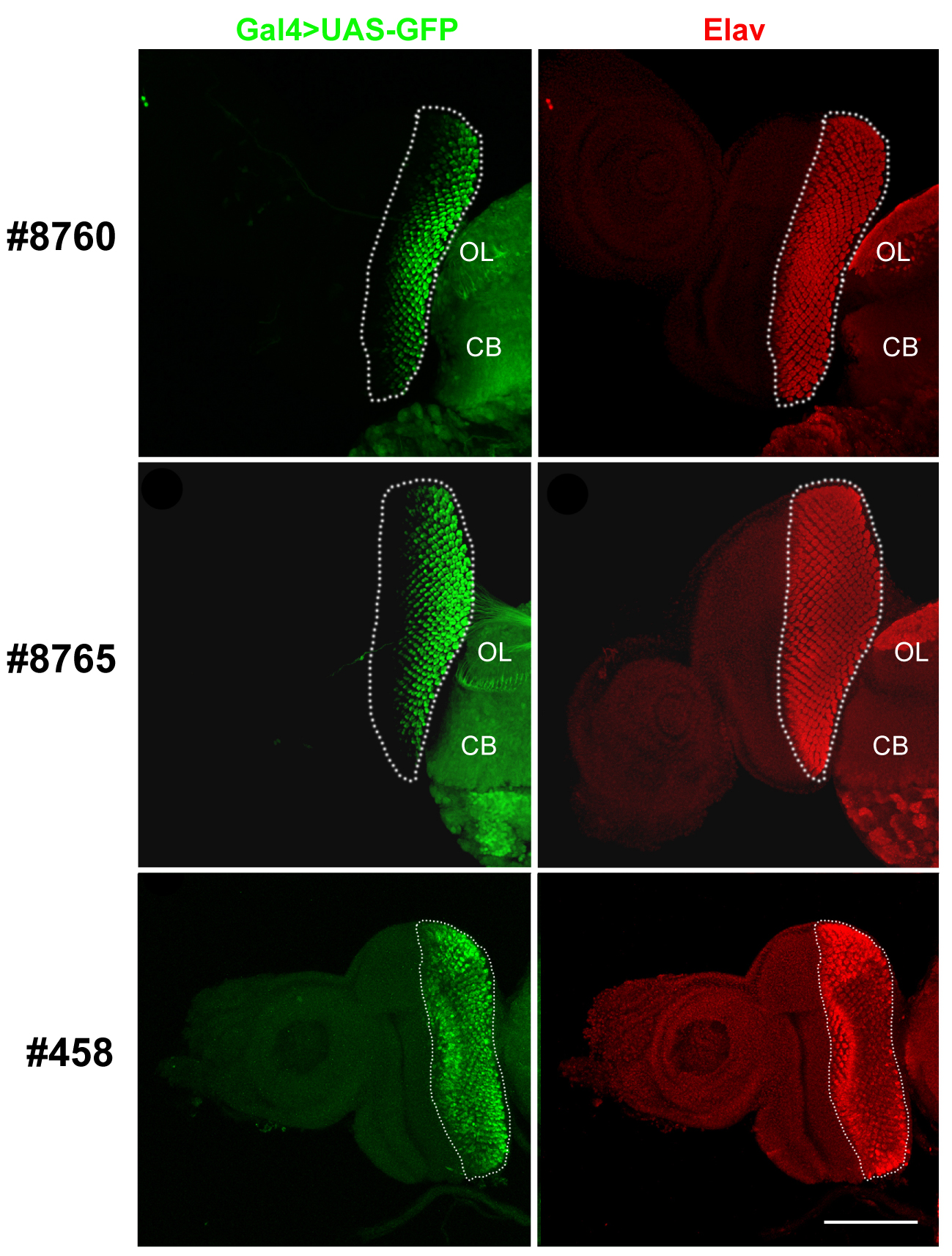

Supplement: Supplementary file 4 — Additional file 4: Figure S3. Comparison of GFP expression patterns elicited by the Gal4 drivers from the BDSC lines #8760, #8765, and #458 with the Elav immunostaining pattern in the eye-antennal imaginal discs from third-instar larvae. CB, central brain; OL, optic lobe. Scale bar: 100 μm. [file 12863_2020_895_MOESM4_ESM.jpg]

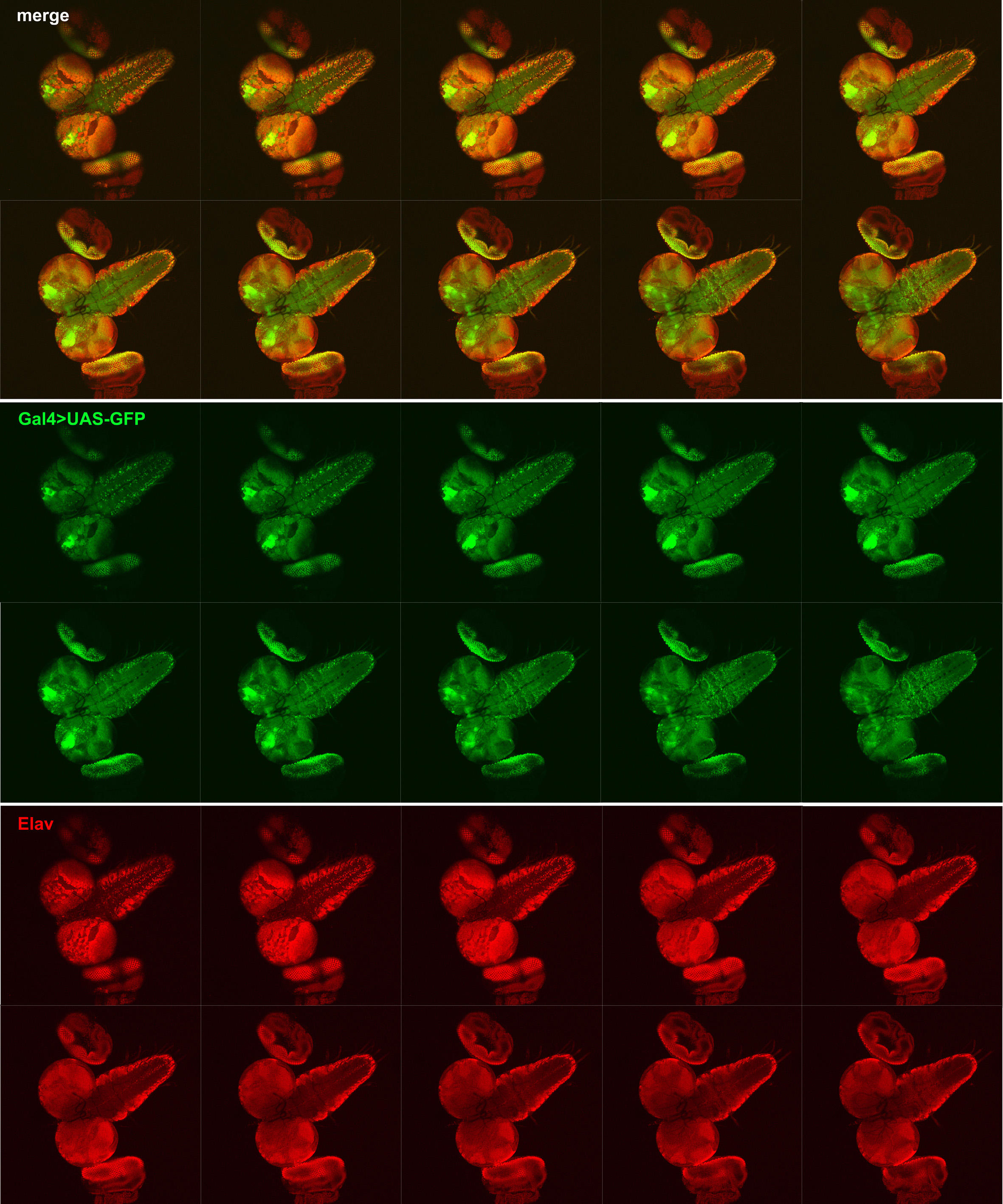

Supplement: Supplementary file 5 — Additional file 5: Figure S4. Representative series of z-stack confocal images of the CNS and eye-antennal imaginal discs from a third-instar larva expressing GFP under the control of the elavC155 driver from the BDSC line #458. The tissues are stained with anti-Elav antibodies. [file 12863_2020_895_MOESM5_ESM.jpg]

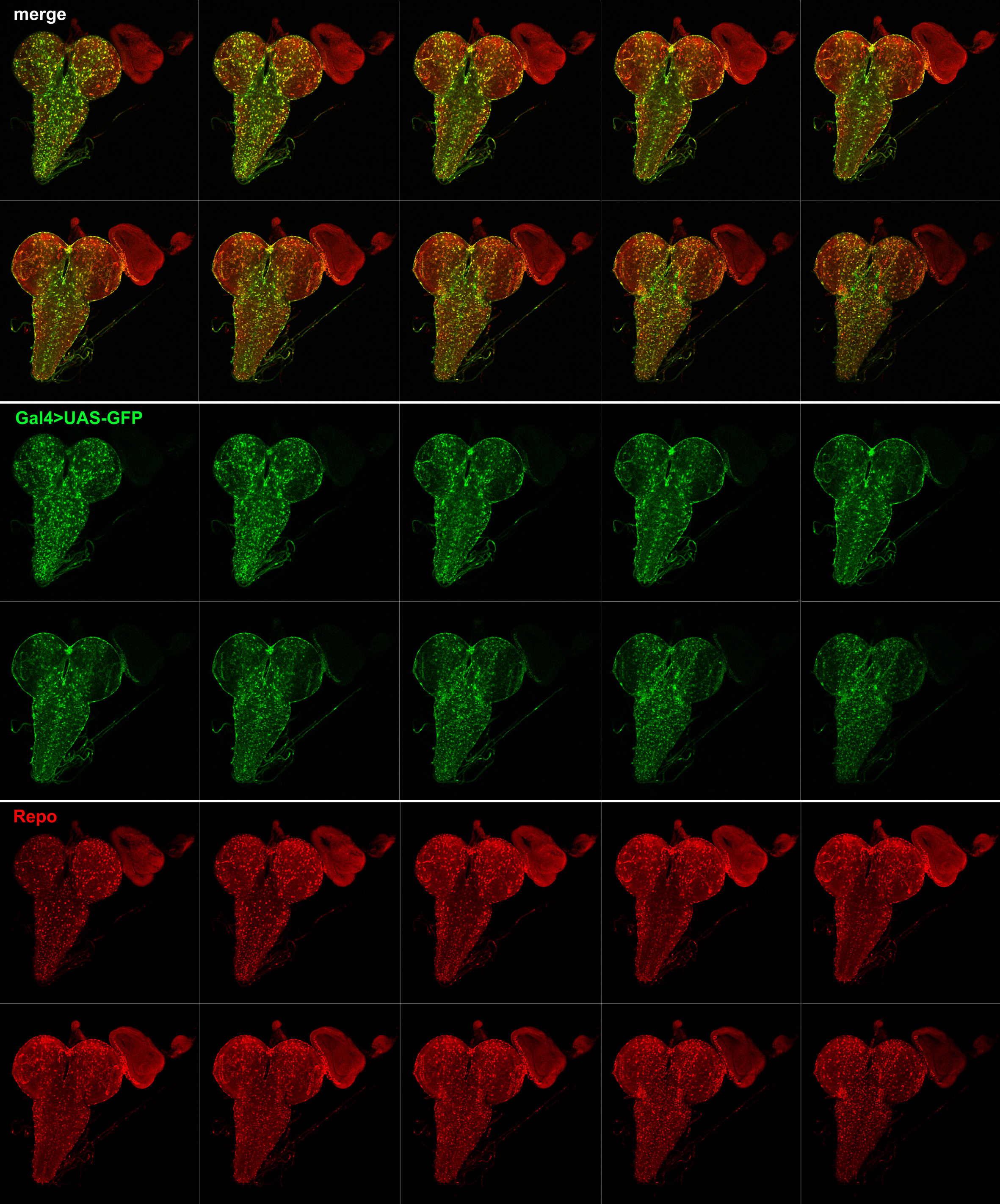

Supplement: Supplementary file 6 — Additional file 6: Figure S5. Representative series of z-stack confocal images of the CNS and eye-antennal imaginal discs from a third-instar larva expressing GFP under the control of the Gal4repo driver from the BDSC line #7415. The tissues are stained with anti-Repo antibodies. [file 12863_2020_895_MOESM6_ESM.jpg]

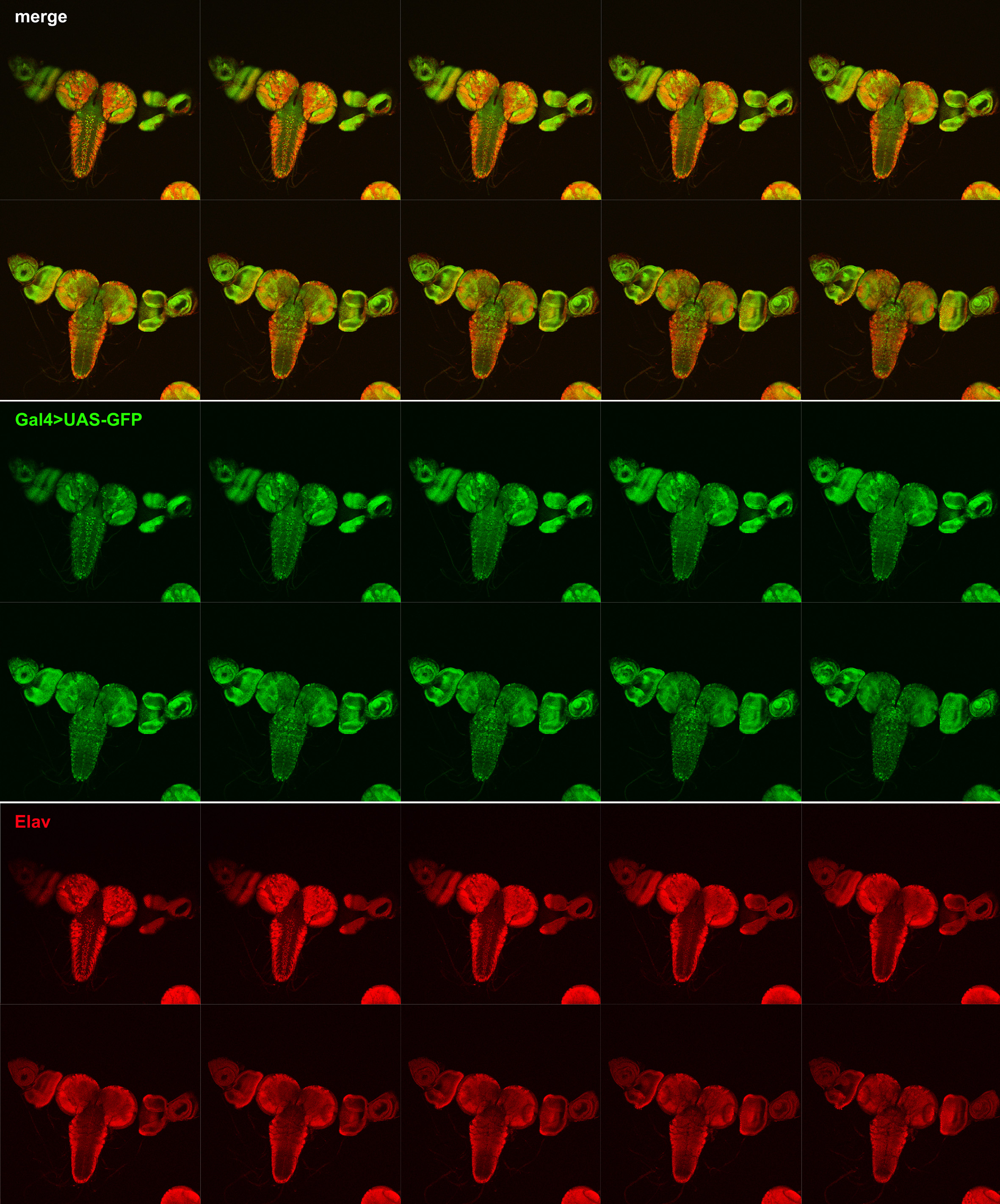

Supplement: Supplementary file 7 — Additional file 7: Figure S6. Representative series of z-stack confocal images of the CNS and eye-antennal imaginal discs from a third-instar larva expressing GFP under the control of the 69B-Gal4 driver from the BDSC line #1774. The tissues are stained with anti-Elav antibodies. [file 12863_2020_895_MOESM7_ESM.jpg]

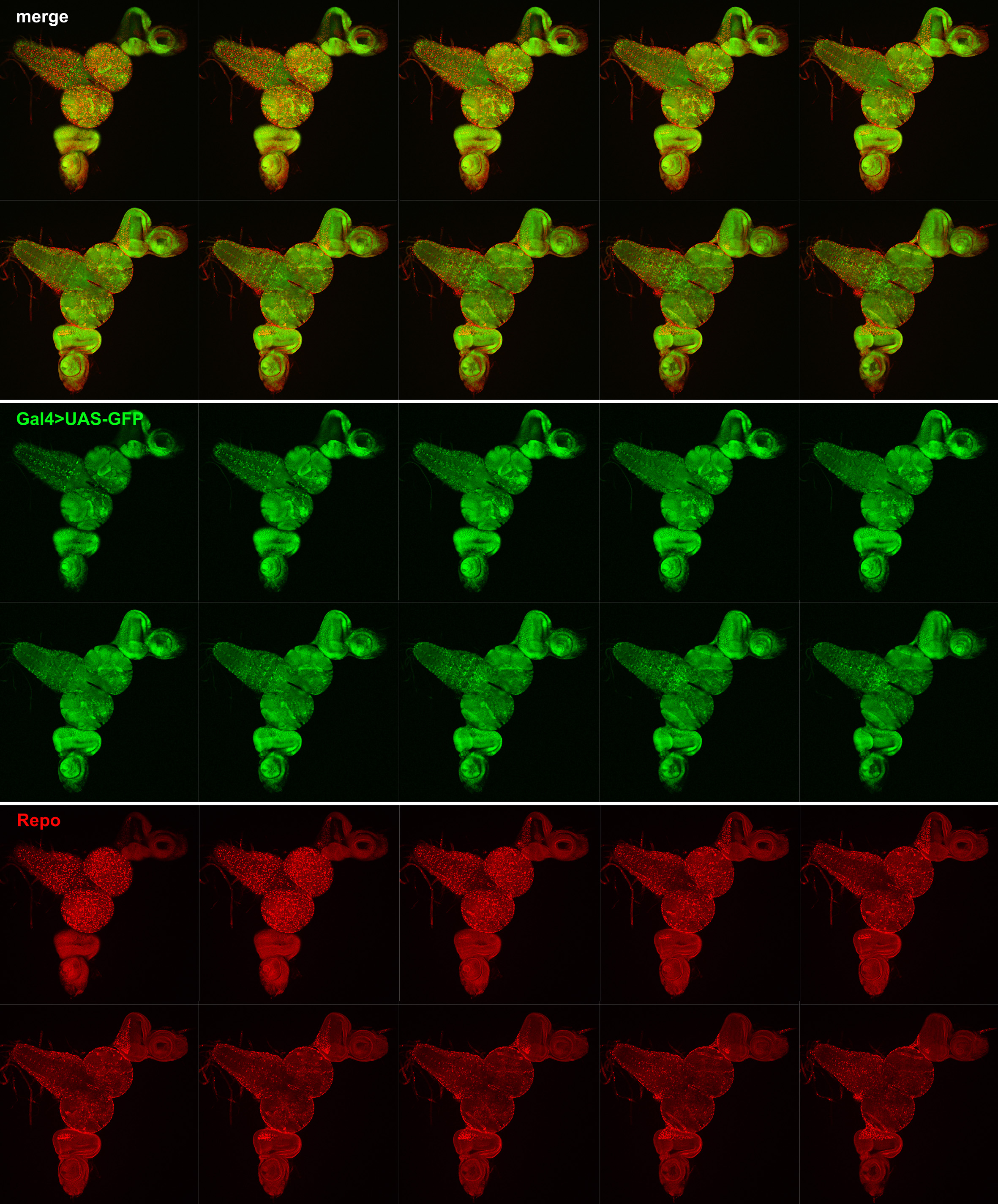

Supplement: Supplementary file 8 — Additional file 8: Figure S7. Representative series of z-stack confocal images of the CNS and eye-antennal imaginal discs from a third-instar larva expressing GFP under the control of the 69B-Gal4 driver from the BDSC line #1774. The tissues are stained with anti-Repo antibodies. [file 12863_2020_895_MOESM8_ESM.jpg]
